# Carbohydrate knowledge, beliefs, and intended practices, of endurance athletes who report exercise-associated gastrointestinal symptoms

**DOI:** 10.3389/fnut.2023.1133022

**Published:** 2023-04-12

**Authors:** Rachel Scrivin, Ricardo J. S. Costa, Fiona Pelly, Dana Lis, Gary Slater

**Affiliations:** ^1^School of Health and Behavioural Sciences, University of the Sunshine Coast, Sippy Downs, QLD, Australia; ^2^Faculty of Health, Education and Environment, Te Pukenga, New Zealand Institute of Skills and Technology, Tauranga, New Zealand; ^3^Department of Nutrition Dietetics and Food, Monash University, Melbourne, VIC, Australia; ^4^Department of Neurobiology, Physiology and Behaviour, University of California, Davis, CA, United States

**Keywords:** exercise-induced gastrointestinal syndrome, athlete preferences, athletic performance, nutrition, prolonged exercise

## Abstract

This study aimed to explore carbohydrate (CHO) knowledge, beliefs, and intended practices of endurance athletes who experience exercise-associated gastrointestinal symptoms (Ex-GIS) compared to those without Ex-GIS. A validated online questionnaire was completed by endurance athletes (*n* = 201) participating in >60 min of exercise that present with Ex-GIS (*n* = 137) or without (*n* = 64). Descriptive statistics were used for parametric and non-parametric data with appropriate significance tests. Associations between categorical data were assessed by Chi-square analysis, and post-hoc Bonferroni tests were applied when significant. A content analysis of open-ended responses was grouped into themes, and quantitative statistics were applied. Participants included runners (*n =* 114, 57%), triathletes (*n* = 43, 21%) and non-running sports (*n* = 44, 21%) who participate in recreational competitive (*n* = 74, 37%), recreational non-competitive (*n* = 64, 32%), or competitive regional, national, or international levels (*n* = 63, 31%). Athletes correctly categorized CHO (*x̄* = 92–95%) and non-CHO (*x̄* = 88–90%) food and drink sources. On a Likert scale of 1 (strongly disagree) to 5 (strongly agree) athletes typically agree or strongly agree that consuming CHO around key training sessions and competitions enhances athletic performance [median = 4 (IQR, 4–5)], and they intend to consume more CHO around exercise [median = 3 (IQR, 2–3)]. No differences in beliefs and intentions were found among athletes with or without Ex-GIS. To enhance athletic performance, most endurance athletes intend to consume more CHO around exercise. Adequate knowledge of CHO-containing food sources was apparent; however, specific CHO ingestion practices remain to be verified.

## Introduction

Endurance athletes frequently report developing gastrointestinal symptoms around exercise, e.g., bloating, urge to defecate, burping, regurgitation, and diarrhea, referred to as exercise-associated gastrointestinal symptoms (Ex-GIS) (1–3). Ex-GIS develop through the gastrointestinal circulatory or neuro-endocrine pathways or *via* mechanical strain on the peritoneal cavity (2, 4). A description of Ex-GIS has been defined within the exercise-induced gastrointestinal syndrome model, outlining the complex pathophysiology involved in the development of Ex-GIS (5, 6). Many endurance athletes who report Ex-GIS implement strategies to manage symptomology (7–9), including reducing CHO-rich foods such as bread, cereals, milk, and yoghurt (7). Therefore, endurance athletes with Ex-GIS may be modifying their dietary intake to reduce Ex-GIS development, perhaps at the expense of their CHO intake. Given the critical role carbohydrates play as a substrate during exercise to support performance enhancement (10, 11), carbohydrate intake guidelines have been established for athletes, including total daily targets, plus specific guidance before, during, and after exercise (12, 13).

Endurance athletes typically train for >60 min several times a week. However, the recommended CHO intake ranges vary depending on the duration and intensity of exercise (12, 13). An individualized approach is recommended for CHO consumption, however as a general guideline of 3 g.kg^−1^.d^−1^ CHO for low-intensity or skill-based training, up to 8–10 g.kg^−1^.d^−1^ CHO for moderate to high-intensity or sustained exercise (12, 13). For key training sessions or competitions, 10–12 g.kg^−1^.d^−1^ CHO, 24–36 h before sustained endurance events, is associated with improved performance (12, 13). During endurance exercise lasting 1–3 h, it is recommended that athletes consume 30–60 g.h^−1^ CHO (12, 13). More extended endurance activities lasting >3 h, 90 g.h^−1^ CHO from multiple transportable CHO sources (i.e., glucose and fructose in a ratio of 2:1) to increase CHO availability and CHO oxidation efficiency, thereby reducing fatigue and improving athletic performance (12, 13). Despite these recommendations, many endurance athletes do not meet daily CHO targets leading up to competition (14) or during endurance events (3, 15, 16), possibly due to a lack of practicing with race day nutrition or experimenting with food and fluid quantities to determine feeding tolerance and preferences (17). Individuals who experience Ex-GIS may be more susceptible to not achieving CHO intake targets due to varied dietary strategies implemented to reduce symptomology (7–9).

Interestingly, athletes that present with Ex-GIS may benefit from the ingestion of CHO during exercise, as this strategy has been shown to reduce the development and severity of Ex-GIS and mitigate disruptions to the integrity of the intestinal epithelium (2, 18–22). CHO consumed during exercise increases portal vein blood supply, enhancing blood flow to the splanchnic region (23). This likely increases intestinal epithelial blood flow, thereby reducing intestinal injury and the subsequent development of gastrointestinal complications (18, 19). However, ingestion of CHO above an individual’s tolerance level may exacerbate Ex-GIS, irrespective of exercise duration or intensity (24).

This exploratory study investigates the CHO knowledge, beliefs, and intended practices of endurance athletes who report Ex-GIS. Due to the complex pathophysiology of the development of Ex-GIS, it is hypothesized that endurance athletes who experience Ex-GIS may have beliefs and intentions about CHO consumption that varies from sports nutrition guidelines or recommendations (10, 12, 13).

## Methods

### Participants

This exploratory study applied a validated online questionnaire investigating endurance athletes who report Ex-GIS and their CHO knowledge, beliefs, and intended practices (25) (Supplementary file 1), compared to a subgroup of athletes without Ex-GIS. Convenience sampling was used for athlete recruitment given researchers had associations with endurance athlete groups or professional contacts who support endurance athletes. Endurance athletes ≥18 years (y) of age, participating in endurance events ≥60 minutes (min), were invited to participate in the study. Only questions related to CHO knowledge, beliefs, intended practices and sports nutrition strategies were included in this study. Specific questions regarding nutritional strategies for managing Ex-GIS, including the use of nutritional supplements were excluded, as this has been previously reported (26).

This study was approved by the Human Research Ethics Committee (University of the Sunshine Coast, Australia), ethics approval number S201402 and conducted in accordance with the Declaration of Helsinki for human research. Athletes accessed the questionnaire online through Qualtrics Core XM survey platform (RRID:SCR_016728) (Qualtrics LLC, 333 West River Park Drive, Provo, UT 84604, United States). Athletes were required to provide written informed consent and all data collected was anonymous. Demographic data collected included biological sex and age. Other information collected included main sport, participation level, and event characteristics (single-day or multi-day).

### Questionnaire

Using a dichotomous scale, 15 food and drink options were categorized as CHO or non-CHO food or drink sources to determine athletes CHO knowledge. Athletes rated their beliefs about consuming CHO for enhancing athletic performance at various time points around exercise on a Likert scale from 1 (strongly disagree) to 5 (strongly agree). The time points assessed were 1–2 days before events lasting ≥90 min, in the last meal or snack before endurance training or events lasting ≥90 min, during endurance exercise or an event lasting ≥60 min, and within 30 min after endurance exercise when there is <8 h between events. Athletes also rated their intention to consume CHO at various time points around exercise on a Likert scale from 1 (intend to eat less), 2 (intend to eat the same) or 3 (intend to eat more). A content analysis was conducted on open-ended responses collected on self-selected nutritional strategies implemented around exercise, which were reviewed by the research team and categorized into themes (27).

### Statistical analysis

To enable comparisons between larger data sets, cycling, swimming, walking, multisport, and other sports were collapsed into a new category called non-running sports. Additional data was collapsed when categories had more than 20% of the cells with less than an expected count of five, e.g., competitive regional, national, and international participation groups were collapsed into a new category, ‘CompRNI’. The Shapiro Wilks test was used to determine normality of data and homogeneity of variances before other appropriate tests were applied. Parametric data were described as the mean ± standard deviation, and for non-parametric data, the median and interquartile range (IQR). For parametric data, continuous variables were analyzed using independent samples t-tests with Cohen’s *d* for effect sizes and the Mann–Whitney U test for non-parametric data. For categorical data, sex, main sport, participation level, and event characteristics, were assessed for associations using Chi-square analysis and Bonferroni post-hoc tests applied when significant. Open-ended responses were categorized into themes, with quantitative frequency statistics applied. Significance was accepted with α ≤0.05. Data were analyzed using IBM SPSS Statistics 27.0 (RRID:SCR_019096) (IBM Corporation New Orchard Road Armonk, NY 10504-1722, United States).

## Results

Two hundred and one endurance athletes completed the validated online questionnaire (25), with 137 reporting Ex-GIS. No differences were found in responses regarding CHO knowledge, beliefs or intended practices in those athletes who also reported a diagnosed gastrointestinal disease or disorder (*n* = 15). Therefore, all responses from this subgroup were collapsed and included in data analysis for those with Ex-GIS.

As this was an exploratory study, a *post-hoc* G*Power (version 3.1.9.7 (RRID:SCR_013726) calculation was used to determine if the desired sample size was attained. A goodness-of-fit test was used with a medium effect size (*w* = 0.3), alpha (α) of 0.05 and total sample size of 201; this determined that adequate power of 0.92 was achieved (28).

### Demographics and event characteristics

When comparing athletes reporting Ex-GIS with those without Ex-GIS, no significant group differences were found for sex and age within groups or between groups (Table 1). The mean age from the Ex-GIS cohort was 42 ± 11 y compared to 40 ± 11 y in those without Ex-GIS (*t*_(129)_ = 1.1, *p* = 0.3, *d* = 0.2). More females with Ex-GIS (*n* = 86, 63%) completed the questionnaire, however this was not significantly different to males (*df* = 1, *x*^2^ = 0.7, *p* = 0.7) (Table 1). After Chi-square analysis, no associations were found between main sport and participation level, event characteristics for both those with Ex-GIS and those without Ex-GIS (Table 2).

**Table 1:** Participant demographics of endurance athletes with (*n* = 137) and without (*n* = 64) reported exercise-associated gastrointestinal symptoms with Chi-square comparisons for categorical data (sex) and independent samples t-test for normally distributed data (age).

		Sex				Age					Totals *n*	F *n*, (%)	M *n*, (%)	Association within group and sex *χ^2^* *(p)*	Association between groups and sex *χ^2^* *(p)*	*F* Mean (± SD)	M Mean (± SD)	Within group Mean (± SD)	Within group Cohen’s *d* (*p*)	Between group Cohen’s *d* (*p*)
With Ex-GIS^a^	137	86 (63)	51 (37)	0.7 (0.7)	2.3 (0.1)	42 (11)	41 (11)	42 (11)	−0.1 (0.5)	0.2 (0.3)
Without Ex-GIS	64	33 (52)	31 (48)	1.5 (0.5)		40 (12)	40 (9)	40 (11)	0.1 (0.8)	

a Ex-GIS, exercise-associated gastrointestinal symptoms.

F, female; M, male; n, number; SD, standard deviation; *p*, probability; % percentage; *χ^2^*, Chi-square statistic.

**Table 2:** Main sport, competition level and event characteristics of endurance athletes with (*n* = 137) and without (*n* = 64) reported exercise-associated gastrointestinal symptoms analyzed with Chi-square comparisons for categorical data.

		Main sport						Competition level				Event characteristic				Totals *n*	Running *n*, (%)	Triathlon *n*, (%)	Non-running sports *n*, (%)	Association within group (with or without Ex-GIS) and main sport *χ^2^* *(p)*	Association between groups (with or without Ex-GIS) and main sport *χ^2^* *(p)*	Rec,^a^ Non-Comp^b^ *n*, (%)	Rec.Comp n, (%)	Comp RNI^c^n, (%)	Association within group (with or without Ex-GIS) and, comp level *χ*^2^ (*p*)	Association between groups (with or without Ex-GIS) and, comp level *χ*^2^ (*p*)	Single Day *n*, (%)	Multi-day ≥2 days *n*, (%)	Association within groups (with or without Ex-GIS) and, event *χ*^2^ (*p*)	Association between groups (with or without Ex-GIS) and, event *χ*^2^ (*p*)
With Ex-GIS^d^	137	75 (55)	30 (22)	32 (23)	0.2 (0.9)	0.6 (0.8)	44 (32)	51 (37)	42 (31)	0.1 (1)	0.3 (0.9)	129 (94)	8 (6)	0.6 (0.8)	1.6 (0.2)
Without Ex-GIS	64	39 (61)	13 (20)	12 (19)	0.5 (0.8)		19 (30)	23 (36)	22 (34)	0.2 (1)		57 (89)	7 (11)	0.8 (0.6)	

a Rec, recreational.

b Comp, Competitive.

c CompRNI, Competitive regional, national and international.

d Ex-GIS, exercise-associated gastrointestinal symptoms.

F, female; M, male; *n*, number; SD, standard deviation; *p*, probability; % percentage; ≥ greater than or equal to, *χ^2^*, Chi-square statistic.

### Carbohydrate knowledge

Athletes with Ex-GIS mostly categorized CHO and non-CHO food and drinks sources correctly, i.e., *x̄* = 95 and 88% respectively, which was similar for athletes without Ex-GIS, i.e., *x̄* = 92% and *x̄* = 90% (Table 3). No other significant differences were found in CHO knowledge between athletes with or without Ex-GIS for main sport, participation level, event characteristics or biological sex.

**Table 3:** Carbohydrate knowledge comparisons between endurance athletes with (*n* = 137) and without (*n* = 64) reported exercise-associated gastrointestinal symptoms for 15 food and drink items classified as either carbohydrate or non-carbohydrate choices analyzed with Chi-squared comparison and post-hoc Bonferroni adjustment.

Foods and Drinks choices	Athletes with Ex-GIS^c^			Athletes without Ex-GIS			Group differences	Total response for CHO^a^, *n (*%)	Total response for non-CHO^b^, *n (*%)	Association between CHO or non-CHO choice, athletes with Ex-GIS *χ^2^* *(p)*	Total response for non-CHO, *n (*%)	Total response for CHO, *n (*%)	Association between CHO or non-CHO choice, athletes without Ex-GIS *χ*^2^ *(p)*	Association between groups with and without Ex-GIS, CHO or non-CHO choices *χ*^2^ *(p)*
*Carbohydrate foods*
2 slices of wholemeal bread	137 (100)	0 (0)	0 (1)	64 (100)	0 (0)	0 (1)	0 (1)
1 cup of raw rolled oats	137 (100)	0 (0)	0 (0.2)	61 (95)	3 (5)	1.4 (0.1)	6.5 (0.01)^†^
1 cup cooked pasta	137 (100)	0 (0)	0 (1)	64 (100)	0 (0)	0 (1)	0 (1)
1 medium baked potato	136 (99)	1 (1)	0.1 (0.7)	64 (100)	0 (0)	0 (0.6)	0.5 (0.5)
1 cup cooked white rice	136 (99)	1 (1)	0.1 (0.7)	64 (100)	0 (0)	0 (0.6)	0.5 (0.5)
2 wheat biscuits (e.g., Weetbix)	133 (97)	4 (3)	0 (1)	62 (97)	2 (3)	0 (1)	0 (1)
1 large banana	128 (93)	9 (7)	5.3 (0.1)	50 (78)	14 (22)	4 (0.01)^†^	10.1 (0.001)^†^
300 ml of sports drink (e.g., Gatorade)	126 (92)	11 (8)	2.2 (0.2)	52 (81)	12 (19)	2.3 (0.1)	5 (0.1)
300 ml soft drink/ soda (e.g., cola)	106 (77)	31 (23)	0 (1)	49 (77)	15 (23)	0 (1)	0 (1)
*Non-carbohydrate foods*
100 g baked salmon	1 (1)	136 (99)	0.1 (0.7)	0 (0)	64 (100)	0 (0.6)	0.5 (0.5)
100 g grilled chicken	5 (4)	132 (96)	0.1 (0.8)	3 (5)	61 (95)	0.1 (0.7)	0.1 (0.7)
2 boiled eggs	11 (8)	126 (92)	0.2 (0.6)	3 (5)	61 (95)	0.8 (0.5)	0.8 (0.4)
1/2 a large avocado	14 (10)	123 (90)	0.2 (0.6)	4 (6)	60 (94)	0.8 (0.5)	0.8 (0.4)
4 large leaves of iceberg lettuce	24 (18)	113 (82)	0 (1)	12 (19)	52 (81)	0 (0.9)	0.1 (0.8)
1 cup raw broccoli	42 (31)	95 (69)	0.3 (0.6)	15 (23)	49 (77)	0.8 (0.4)	1.1 (0.3)

a CHO, carbohydrate.

b non-CHO, non-carbohydrate.

c Ex-GIS, exercise-associated gastrointestinal symptoms.

g, grams; mL, milliliter; *n*, number; *p*, probability value; *χ^2^* chi-square statistic; % Percentage,

† *p* < 0.05 significant after post-hoc Bonferroni adjustment.

### Carbohydrate beliefs and intended practices

**Figure 1:**
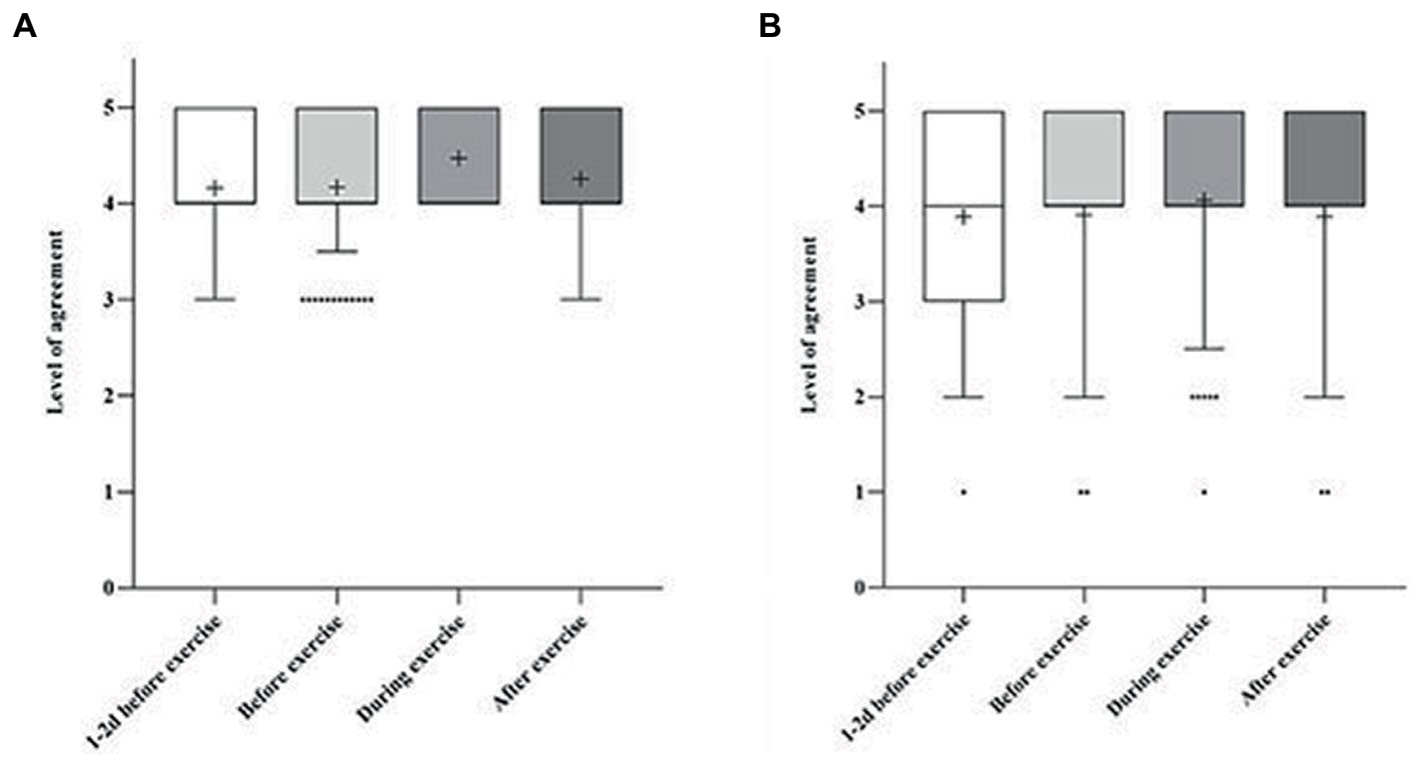
Box and whisker chart showing the median and 10–90th percentile range for endurance athletes with (*n* = 137) and without (*n* = 64) exercise-associated gastrointestinal symptoms (Ex-GIS) and their beliefs about consuming carbohydrates before, during, and after exercise in relation to enhancing athletic performance (i.e., timing of carbohydrate consumption). Data violates homogeneity of variances and is not normally distributed. Outliers are included to show variation in responses. Athletes’ beliefs about consuming carbohydrates to enhance performance are measured by the athlete’s level of agreement with each statement using a Likert scale, 1, strongly disagree; 2, disagree; 3, neither agree nor disagree; 4, agree; 5, strongly agree. Timing defined as: 1–2d before exercise = 1–2 days before an event ≥90 min; Before exercise = in last meal or snack before endurance training or an event ≥90 min; During exercise = during endurance training or an event ≥60 min; After exercise = within 30 min after endurance exercise when there is ≤8 h between sessions; **(A)**, athletes with reported Ex-GIS; **(B)**, athletes without reported Ex-GIS; + mean; • outlier.

**Figure 2:**
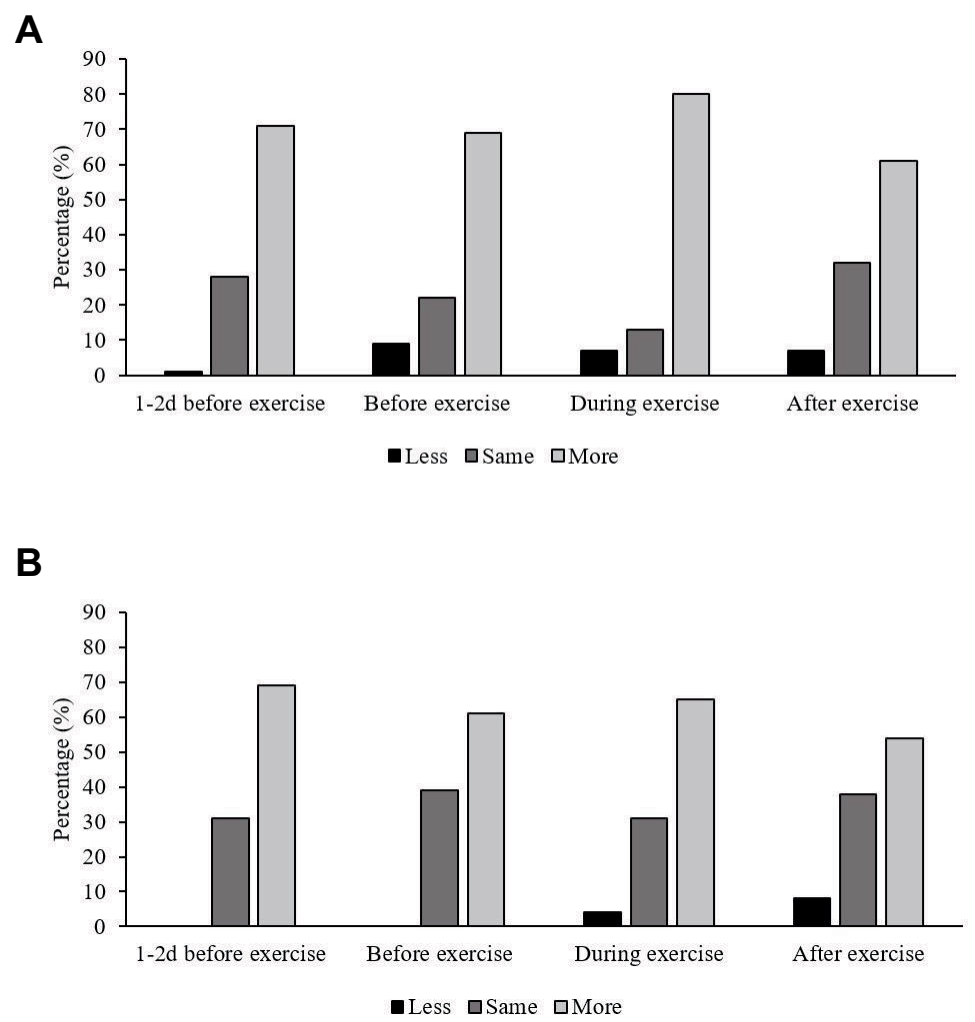
Bar chart showing endurance athletes with (*n* = 137) and without (*n* = 64) exercise-associated gastrointestinal symptoms (Ex-GIS) and their intended practices for consuming less, the same or more carbohydrates around various exercise times. Intention to consume carbohydrates is indicated by a Likert scale: 1, intention to consume less; 2, intention to consume the same; 3, intention to consume more. Timing defined as: 1–2 d before exercise = 1–2 days before an event ≥90 min; Before exercise = in last meal or snack before endurance training or an event ≥90 min; During exercise = during endurance training or an event ≥60 min; After exercise = within 30 min after endurance exercise when there is ≤8 h between sessions; **(A)** athletes with reported Ex-GIS; **(B)**, athletes without reported Ex-GIS.

The majority of athletes *strongly agreed (5 on the Likert scale)* or *agreed (4 on the Likert scale)* with consuming CHO around endurance exercise to enhance athletic performance at all time points assessed (Figure 1). There was no difference found between CHO beliefs in athletes with or without Ex-GIS for all time points assessed (Table 4). Athletes also intended to consume more CHO at all time points assessed (Figure 2), with median scores of 3 (IQR 2–3), and no differences were evident within and between groups (i.e., those with and without Ex-GIS) (Table 5).

**Table 4:** Endurance athletes with (*n* = 136–137) and without exercise-associated gastrointestinal symptoms (*n* = 64) beliefs surrounding the timing of carbohydrate consumption around endurance exercise, measured by the level of agreement using a five point Likert scale from strongly disagree to strongly agree, analyzed with Chi-squared comparison with post-hoc Bonferroni adjustment.

Timing of carbohydrate consumption	Athletes with Ex-GIS^a^ *n*, (%)							Athletes without Ex-GIS *n*, (%)								Total, *n*	Strongly disagree	Disagree	Neither agree nor disagree	Agree	Strongly agree	Assoc^b^ between agreement responses, *x*^2^*(p)*	Total, *n*	Strongly disagree	Disagree	Neither agree nor disagree	Agree	Strongly agree	Assoc. between agreement responses, *x*^2^*(p)*	Assoc. between athletes with and without Ex-GIS, *x*^2^*(p)*
1–2 d before exercise	137	2 (2)	11 (8)	15 (11)	74 (54)	35 (25)	0.5 (1.0)	64	1 (2)	6 (9)	10 (16)	29 (45)	18 (28)	1 (0.9)	1.6 (0.8)
Before exercise	136	0 (0)	12 (9)	12 (9)	79 (58)	33 (24)	0.2 (0.8)	64	2 (3)	5 (8)	7 (11)	33 (52)	17 (26)	1.4 (0.4)	4.9 (0.3)
During exercise	137	1 (1)	6 (4)	12 (9)	62 (45)	56 (41)	1.2 (1)	64	1 (2)	5 (8)	9 (14)	23 (35)	26 (41)	1.9 (0.7)	3.3 (0.9)
After exercise	136	1 (1)	8 (6)	13 (9)	68 (50)	46 (34)	1.4 (1)	64	2 (3)	5 (8)	8 (12)	32 (50)	17 (27)	1.4 (0.8)	2.9 (0.6)

a Ex-GIS, exercise-associated gastrointestinal symptoms.

b Assoc, association.

d, day; *n*, number; *p*, probability; *x*^2^, chi-square statistic; Timing defined as 1-2d before exercise = 1–2 days before an event ≥90 min; Before exercise = in last meal or snack before endurance training or an event ≥90 min; During exercise = during endurance training or an event ≥60 min; After exercise = within 30 min after endurance exercise when there is ≤8 h between sessions.

**Table 5:** Endurance athletes intentions to consume carbohydrate around exercise measured as the level of agreement using a three point Likert scale from eat less to eat more, for athletes with (*n* = 87) or without (*n* = 26) exercise-associated gastrointestinal symptoms.

	Athletes with Ex-GIS^a^ *n*, (%)					Athletes without Ex-GIS^a^ *n*, (%)					
Timing of carbohydrate consumption around endurance exercise	Total, *n*	Less CHO^b^	Same CHO	More CHO	Assoc^c^ between intention to consume CHO, *x*^2^*(p)*	Total, *n*	Less CHO	Same CHO	More CHO	Assoc. between intention to consume CHO, *x*^2^*(p)*	Assoc. between athletes with and without Ex-GIS, and intention to consume CHO, *x*^2^*(p)*
1–2 days before exercise	87	1 (1)	24 (28)	62 (71)	0.1 (1)	26	0 (0)	8 (31)	18 (69)	0.1 (0.9)	0.4 (0.8)
Before exercise	87	8 (9)	19 (22)	60 (69)	1 (0.6)	26	0 (0)	10 (39)	16 (61)	1.2 (0.2)	4.7 (0.1)
During exercise	87	6 (7)	11 (13)	70 (80)	1.4 (0.6)	26	1 (4)	8 (31)	17 (65)	2.5 (0.2)	4.8 (0.1)
After exercise	87	6 (7)	28 (32)	53 (61)	0.1 (1)	26	2 (8)	10 (38)	14 (54)	0.3 (0.8)	0.4 (0.8)

a Ex-GIS, exercise-associated gastrointestinal symptoms.

b CHO, carbohydrate.

c Assoc, association.

d, day; *n*, number; *p*, probability; *x*^2^, chi-square statistic; Less CHO, athletes plan to eat less CHO around endurance exercise; Same CHO, athletes plan to eat the same amount of CHO around endurance exercise; More CHO, athletes plan to eat more CHO around endurance exercise; Timing defined as: 1–2 d before exercise = 1–2 days before an event ≥90 min; Before exercise = in last meal or snack before endurance training or an event ≥90 min; During exercise = during endurance training or an event ≥60 min; After exercise = within 30 min after endurance exercise when there is ≤8 h between sessions.

### Dietary strategies

**Figure 3:**
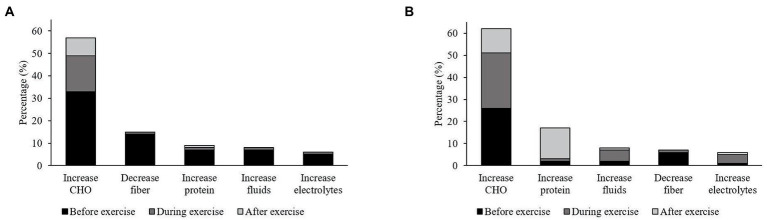
Five most commonly reported responses by endurance athletes with and without exercise-associated gastrointestinal symptoms (Ex-GIS) and the specific nutrition strategies applied (i.e., changes to usual eating habits or changes to the types of foods and drinks usually consumed) before (not further defined by athletes), during, or after endurance training or an event. CHO = carbohydrate. **(A)**, athletes with reported Ex-GIS; **(B)**, athletes without reported Ex-GIS.

Seventy-five percent (*n* = 103) of athletes reported implementing specific nutrition strategies around exercise. The content analysis of the top five open-ended responses was to increase CHO, reduce dietary fiber, and to increase protein, fluid and electrolytes, which were similarly reported in athletes with and without Ex-GIS (Figure 3). The most commonly reported dietary strategy was to increase CHO (*n* = 139, 58%) in both athlete groups, i.e., with Ex-GIS (*n* = 87, 57%) or without Ex-GIS (*n* = 52, 61%), particularly before exercise (Figure 3).

## Discussion

This exploratory study aimed to investigate the CHO knowledge, beliefs, and intended practices of endurance athletes who report Ex-GIS. The main results from the study indicate that endurance athletes with reported Ex-GIS have similar carbohydrate knowledge scores, beliefs, intentions and nutrition strategies as endurance athletes without Ex-GIS. In contrast to our hypothesis, the majority of endurance athletes who report Ex-GIS strongly agreed or agreed that CHO consumed around exercise enhances athletic performance, and they also intend to consume more CHO around exercise to improve their athletic performance. Through a content analysis of open-ended responses, the most common self-reported dietary strategy implemented around exercise was increasing CHO intake, particularly before endurance exercise. These findings broadly align with recommended sports nutrition CHO guidelines (10, 12, 13); however, further research is required to determine specific CHO amounts endurance athletes with Ex-GIS plan to consume around exercise. It is acknowledged that when determining specific CHO intakes during exercise, an individualized approach is advocated to gauge the upper limits of whole-body CHO oxidation rates alongside glucose availability, Ex-GIS and feeding tolerance (24, 29).

In the current investigation, it has been established that endurance athletes with reported Ex-GIS appear to have the intent and CHO knowledge to increase their daily CHO intake in preparation for, during, and in the recovery period after exercise. However, this is not always the case. Findings from a recent investigation that used a validated questionnaire to determine endurance athletes’ knowledge of competition CHO guidelines show athletes scored less than 50% of total correct responses from five areas (30). The five areas investigated were knowledge about CHO storage and metabolism, pre-competition CHO loading, and CHO consumption before, during, and after competition (30). When comparing studies, confirming endurance athletes’ current sports nutrition knowledge level can be challenging due to the heterogeneity between questionnaires, e.g., questionnaire design, specific questionnaire tools used, or target population (31). Athletes with Ex-GIS likely require a greater level of nutrition knowledge to increase CHO intakes while moderating factors that may exacerbate Ex-GIS, such as dietary fiber or fermentable oligo-, di-, monosaccharide and polyols. Athletes may seek the support of an accredited practicing dietitian to assist them in negotiating the complex interaction that can result when attempting to increase carbohydrates for endurance exercise while moderating factors known to exacerbate Ex-GIS. Accredited dietitians are a preferred source of nutrition information for athletes with Ex-GIS (7); however, as with this study, it is likely that due to convenience sampling, athletes may have had prior exposure to dietetic expertise and therefore were more likely to understand fuel mechanics for endurance exercise, thereby resulting in high CHO knowledge scores.

Increasing CHO around endurance exercise was the most commonly self-reported dietary strategy. It is well-documented that consuming CHO around exercise improves athletic performance, reduces fatigue and facilitates muscle glycogen restoration after exercise (10, 12, 13). Furthermore, consuming CHO before and during exercise ameliorates intestinal epithelial damage and permeability during exercise (21), leading to less reported Ex-GIS (18). Observational field studies on endurance athletes without Ex-GIS have also found that CHO consumed during endurance events had no or minimal relationship with the development of gastrointestinal symptoms (1, 14, 16) or performance times (32). However, most athletes were not consuming recommended CHO targets of up to 90 g.h^−1^ CHO for endurance exercise >3 h (1, 15, 16, 32).

It is likely that to improve gastrointestinal tolerance (i.e., less Ex-GIS development) when consuming CHO during exercise, athletes may require a period of gut training (19, 20, 24). Studies implementing a gut-training protocol have shown less reported Ex-GIS and improved performance times (19, 20). Therefore, in an athlete’s nutritional plan, a period of gut training may be warranted to reduce Ex-GIS development during exercise and benefit from higher CHO oxidation rates due to greater exogenous CHO availability (19, 20).

The current study did not investigate the quantification of CHO intake among endurance athletes with Ex-GIS. Athletes have indicated they intend to increase their CHO intake around exercise to enhance performance. However, it is still unknown if they meet the recommended CHO targets for endurance training and recovery; as previous research indicates that many endurance athletes without pre-existing Ex-GIS are not meeting the recommended CHO targets (1, 15, 16, 33). This information would be valuable for practitioners when providing nutrition education and meal planning around CHO intakes to support athletic performance.

## Data availability statement

The raw data supporting the conclusions of this article will be made available by the authors, without undue reservation.

## Ethics statement

The studies involving human participants were reviewed and approved by Human Research Ethics Committee (University of the Sunshine Coast, Australia), ethics approval number S201402. The patients/participants provided their written informed consent to participate in this study.

## Author contributions

RS and GS: conceptualization and writing—original draft. RS, GS, RC, FP, and DL: methodology and writing—review and editing. RS: validation, formal analysis, investigation, and project administration. GS: supervision. All authors contributed to the article and approved the submitted version.

## Funding

The work was supported by the University of the Sunshine Coast, Australia.

## Conflict of interest

The authors declare that the research was conducted in the absence of any commercial or financial relationships that could be construed as a potential conflict of interest.

## Publisher’s note

All claims expressed in this article are solely those of the authors and do not necessarily represent those of their affiliated organizations, or those of the publisher, the editors and the reviewers. Any product that may be evaluated in this article, or claim that may be made by its manufacturer, is not guaranteed or endorsed by the publisher.
